# Ageing affects chondroitin sulfates and their synthetic enzymes in the intervertebral disc

**DOI:** 10.1038/sigtrans.2017.49

**Published:** 2017-09-22

**Authors:** Estelle C Collin, Oliver Carroll, Michelle Kilcoyne, Marianna Peroglio, Eugene See, Doris Hendig, Mauro Alini, Sibylle Grad, Abhay Pandit

**Affiliations:** 1 Centre for Research in Medical Devices (CÚRAM), National University of Ireland, Galway, Ireland; 2 Department of Microbiology, School of Natural Sciences, National University of Ireland, Galway, Ireland; 3 Glycoscience Group, National University of Ireland, Galway, Ireland; 4 AO Research Institute, Davos, Switzerland; 5 Institut für Laboratoriums-und Transfusionsmedizin, Herz-und Diabeteszentrum Nordrhein-Westfalen, Universitätsklinik der Ruhr-Universität Bochum, Bad Oeynhausen, Germany

## Abstract

The depletion of chondroitin sulfates (CSs) within the intervertebral disc (IVD) during degenerative disc disease (DDD) results in a decrease in tissue hydration, a loss of fluid movement, cell apoptosis, a loss of nerve growth inhibition and ultimately, the loss of disc function. To date, little is known with regards to the structure and content of chondroitin sulfates (CSs) during IVD ageing. The behavior of glycosaminoglycans (GAGs), specifically CSs, as well as xylosyltransferase I (XT-I) and glucuronyltransferase I (GT-I), two key enzymes involved in CS synthesis as a primer of glycosaminoglycan (GAG) chain elongation and GAG synthesis in the nucleus pulposus (NP), respectively, were evaluated in a bovine ageing IVD model. Here, we showed significant changes in the composition of GAGs during the disc ageing process (6-month-old, 2-year-old and 8-year-old IVDs representing the immature to mature skeleton). The CS quantity and composition of annulus fibrosus (AF) and NP were determined. The expression of both XT-I and GT-I was detected using immunohistochemistry. A significant decrease in GAGs was observed during the ageing process. CSs are affected at both the structural and quantitative levels with important changes in sulfation observed upon maturity, which correlated with a decrease in the expression of both XT-I and GT-I. A progressive switch of the sulfation profile was noted in both NP and AF tissues from 6 months to 8 years. These changes give an appreciation of the potential impact of CSs on the disc biology and the development of therapeutic approaches for disc regeneration and repair.

## Introduction

Neck and lower back pain are the two greatest causes of job-related disability with significant associated social and economic costs,^1^ accounting for total annual healthcare costs estimated at £11 billion in the UK in 2000^(ref. 2)^ and between $50 to $90 billion each year in the USA.^3^ Strongly associated with intervertebral disc (IVD) degeneration,^4^ these pains are defined epidemiologically as ‘a process related to normal ageing as well as changes related to physical loading over a lifetime’.^5^ The distinction between disc degeneration and the normal ageing process of the IVD remains unclear. Nevertheless, IVD degeneration occurs irrespective of the ageing process because of external factors (for example, environmental and genetic factors).^6,7^

Both the degeneration and ageing of the disc are characterized by an important catabolism of the IVD extracellular matrix (ECM), resulting in the loss of mechanical function.^4,6,8^ The degradation of the ECM is induced by many pro-inflammatory factors such as interleukin-1 beta (IL-1β) and tumor necrosis factor alpha (TNF-α).^9,10^ These two cytokines have been suggested to activate the production of matrix-degrading enzymes such as matrix metalloproteinase-7 (MMP-7), MMP-13 and a disintegrin and metalloproteinase with thrombospondin motif (ADAMTS)-4, inducing the degradation of collagen and proteoglycans (PGs).^9,11^ PGs are glycoproteins on which glycosaminoglycan (GAG) chains are attached. The family of GAGs includes heparan sulfate (HS), chondroitin sulfate (CS), dermatan sulfate (DS) and keratan sulfate (KS).^12^ Their polyanionic nature contributes to their biological functions by interacting with many cytokines, receptors, growth factors and extracellular molecules.^13,14^ Aggrecan is the predominant PG present in the ECM of IVD tissue (15–20% of the annulus fibrosus (AF) and 65% of the nucleus pulposus (NP) dry weight).^6,15–17^ The functional properties of aggrecan are due to the high content of chondroitin sulfates on the molecule and its ability to create aggregates with molecules of hyaluronan (HA), which provide mechanical strength and high hydrodynamic capabilities to the IVD tissue.^6,8,18–21^ During both the IVD degeneration and the ageing process, a change in the structure of GAG chains occurs. This phenomenon leads to the formation of an aggrecan molecule with fewer and shorter CS chains and more KS chains.^21,22^ The depletion of CS chains results in a decrease in tissue hydration,^6^ resulting in a loss of fluid movement,^20,23–25^ cell apoptosis,^4,24,26^ a loss of nerve growth inhibition^27–29^ and, ultimately, the loss of disc function.^4,15,30^

It has been shown that cell behavior in many tissues is not only affected by the structure of CSs but also by changes in their sulfation pattern.^14^ The chondroitin 4-sulfate (C4S) and chondroitin 6-sulfate (C6S) as well as chondroitin 2,6-sulfate and chondroitin 4,6-sulfate disaccharides provide biological function to CS chains by influencing cell signaling or growth factor interaction and by modifying the PG conformation.^14,31^ Although many studies have highlighted the importance of CSs in tissue development and pathologies, little is known about the structure and content of CSs. Important variations in the sulfation patterns of CSs have been reported during embryonic development^32^ and maturation.^33^ However, no study has reported on CS compositional changes upon ageing.

**Figure 1:**
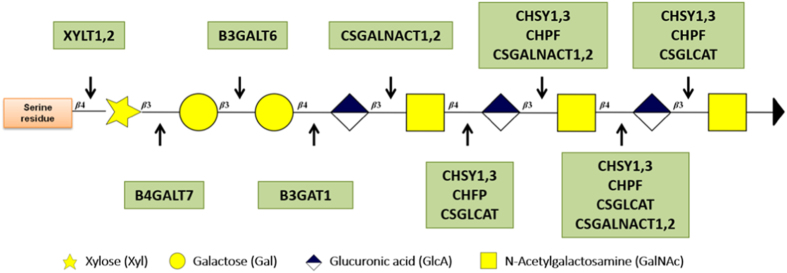
Schematic of CS synthesis [adapted from Laboratories K. KEGG Pathway Maps: Glycosaminoglycans Synthesis, Japan, 2013]. The CS results from the polymerization of a succession of glycans on the serine residue of the Asn-X-Ser/Thr peptidic motif *via* different enzymatic reactions. The enzymes are represented in green, while the protein core is represented in orange.

The synthesis of CS chains is initiated by a tetrasaccharide Xyl-Gal-Gal-GlcA (where Xyl, Gal and GlcA indicate xylose, galactose and glucuronic acid, respectively) attached to the Asn-X-Ser/Thr (asparagine-X-serine/threonine) peptidic sequences of the protein core. The attachment of the initial glycan is catalyzed in the endoplasmic reticulum by xylosyltransferase I (XT-I) and XT-II enzymes.^34^ XT-I, a rate-limiting enzyme,^35^ is considered to be a key regulatory factor of GAG synthesis because of its role as a primer for chain elongation and its tight regulation of the biosynthetic pathway.^34,36,37^ The expression of XT-I is, as with aggrecan, induced by transforming growth factor beta 1 (TGF-β1) via extracellular signal-regulated kinase1/2 (ERK1/2) and p38 mitogen-activated protein kinase (MAPK) pathways.^38^ A decrease of XT-I has been noted during osteoarthritis. This decrease is directly mediated by IL-1β, which inhibits the XT-I promoter by promoting Sp3 transcription factor production.^36^ XT-I expression and its effects have not yet been reported in the IVD tissue. After the transfer of the protein in the golgi apparatus, a succession of glucuronic acid (GlcA) and *N*-acetylgalactosamine (GalNAc) ((GlcA-β*-*(1→3)-GalNAc-β*-*(1→4))_*n*_) glycans^39^ are polymerized on the previously attached tetrasaccharide through the successive activity of glycosyltransferases.^40,41^ β-1,3-glucuronyltransferase-I (GT-I) plays a key role in GAG synthesis as the branching point of various GAG chains. This enzyme catalyzes the transfer of a glucuronyl moiety from a UDP-glucuronic acid onto the galactose of the initial tetrasaccharide^42^ (Figure 1). Responsive to TGF-β, bone morphogenetic protein 2 (BMP-2) and tonicity-responsive enhancer binding protein (TonEBP),^43,44^ the expression of GT-I, like that of XT-I, is down-regulated by IL-1β, leading to a decrease of CS synthesis.^42^ This enzyme has been implicated in CS synthesis in cartilage and IVD tissues.^42,43^

Herein, the behavior of GAGs, specifically CSs, as well as both XT-I and GT-I enzymes were evaluated in a bovine ageing IVD model. It was hypothesized that the expression of both enzymes would correlate with the behavior of GAGs within AF and NP tissues upon ageing. Important changes in the GAG composition during disc ageing were highlighted in this study including the following: decreases of GAGs and CSs were observed upon maturity, which correlated with decreases in the expressions of both XT-I and GT-I. This study highlights the importance of understanding the role of GAGs, especially CSs, to elucidate a better understanding of IVD biology.

## Results

### GAGs, XT-I and GT-I expression

**Figure 2:**
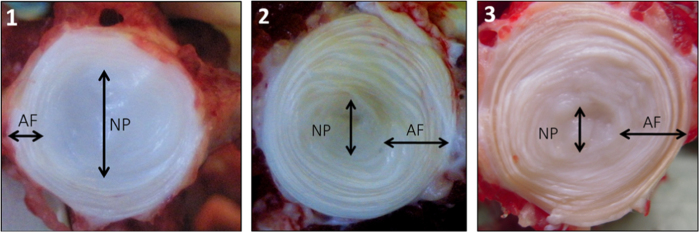
Gross morphologies of 6-month-old (1), 2-year-old (2), and 8-year-old (3) bovine caudal intervertebral discs. A decrease of the NP/AF ratio is observed upon ageing.

**Figure 3:**
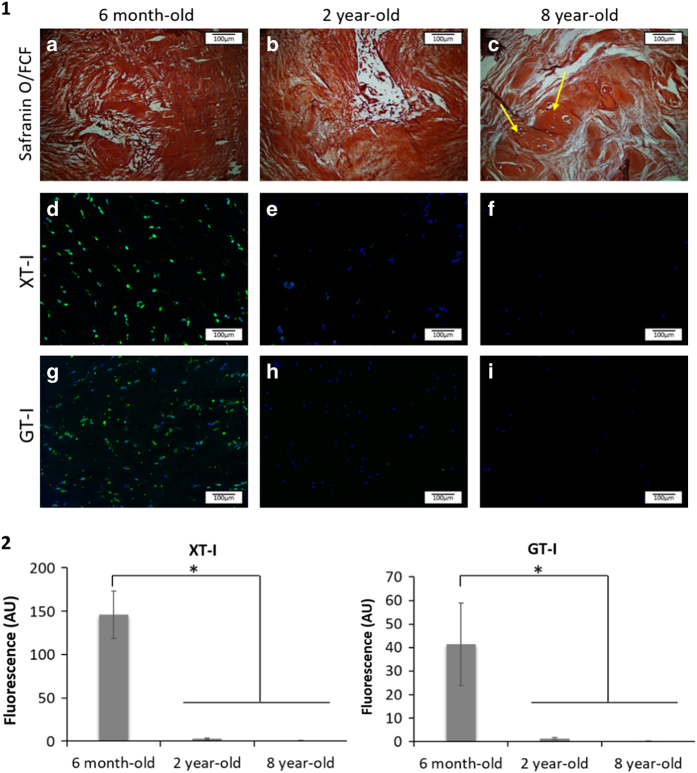
(1) Representative images of nucleus pulposus tissue stained with (**a**–**c**) Safranin O/ fast green (FCF), (**d**–**f**) for XT-I, and (**g**–**i**) for GT-I in 6-month-old (**a**, **d** and **g**), 2-year-old (**b**, **e** and **h**) and 8-year-old (**c**, **f** and **i**) bovine IVD tissues (*n*=5; scale bar=100 μm). (2) Quantification of the XT-I and GT-I staining intensity in NP tissue. Data were normalized to the surface area and are represented as the mean±s.e.m. (*n*=5). * denotes significant differences between the different groups at *P*<0.05.

**Figure 4:**
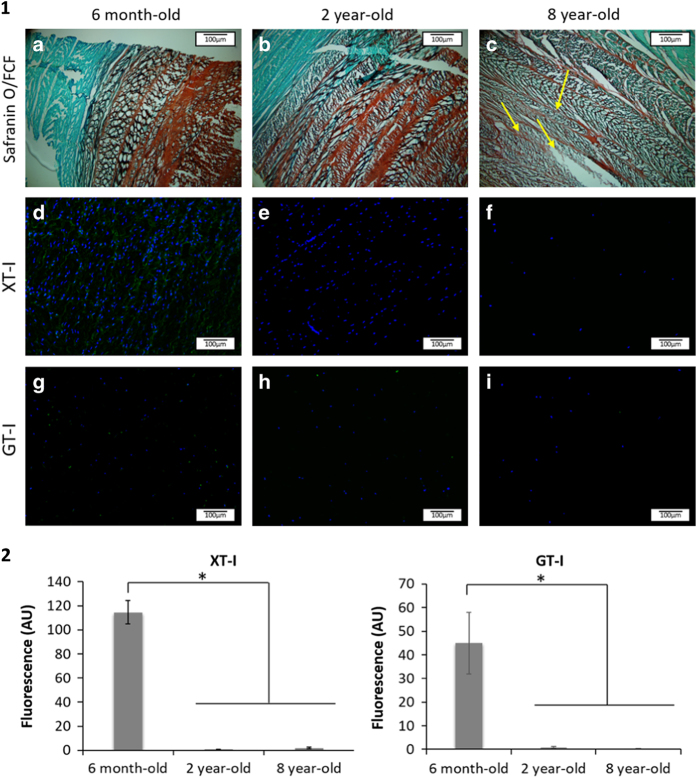
(1) Representative images of AF tissue stained with (**a**–**c**) Safranin O/FCF; (**d**–**f**) for XT-I and (**g**–**i**) for GT-I in 6-month-old (**a**, **d** and **g**), two year-old (**b**, **e** and **h**), and 8-year-old (**c**, **f** and **i**) bovine IVD tissues (*n*=5; scale bar=100 μm). (2) Quantification of XT-I and GT-I staining intensities in AF tissue. Data were normalized to the surface area and are represented as the mean±standard error of the mean (*n*=5). * denotes significant differences between the different groups at *P*<0.05.

Bovine IVDs exhibited different gross morphologies upon ageing (Figure 2). Indeed, with ageing, the tissue appeared less hydrated and more fibrous. The surface area of NP tissue decreased greatly with ageing with increasing ratios of AF surface/NP surface (2.08, 11.64 and 14.3, respectively). Along with these gross morphological differences, the ECM displayed distinct morphological features with ageing. An iterative decrease of GAG staining intensity (Safranin O-Red) was observed in both NP and AF tissues (Figure 3(1) (a–c) and Figure 4(1) (a–c)). This decrease was associated with a change in the ECM organization that was profound in 8-year-old tissue (Figure 3(1) (c) and Figure 4 (1) (c)). The 8-year-old IVD tissues presented an increase in cellularity with characteristic clusters of multiple chondrocyte-like cells (Figure 4(1) (c), arrows). These clusters were delimited by a rim of matrix with a higher staining intensity. An irregular GAG deposition in NP tissue along with a disorganization of the collagen fibers in the AF tissue was observed. An increase in cellularity was observed in both tissues (Figure 3(1) (c) and Figure 4(1) (c)). Annular tears were observed in the AF tissue (Figure 4(1) (c)).

### Decreases of XT-I and GT-I expression

XT-I and GT-I, two enzymes involved in the synthesis of CSs, were stained by immunohistochemistry in order to study their expression as a function of age. XT-I was detected in both NP and AF tissues at the cellular level. No expression of the enzyme was detected in the ECM. XT-I expression decreased significantly in 2-year-old tissues, while the enzyme was no longer detected in 8 year-old IVD tissues (Figure 3 (1) (d–f) and Figure 4(1) (d–f)). Likewise, GT-I was detected in both AF and NP tissues at the cellular level. No expression of the enzyme was detected within the ECM. A significant drop in GT-I expression was observed in IVD tissue as a function of age (Figure 3). GT-1 was not detected in 8-year-old tissue (Figure 3(1) (g–i) and Figure 4(1) (g–i)).

### GAG and CS quantification

**Figure 5:**
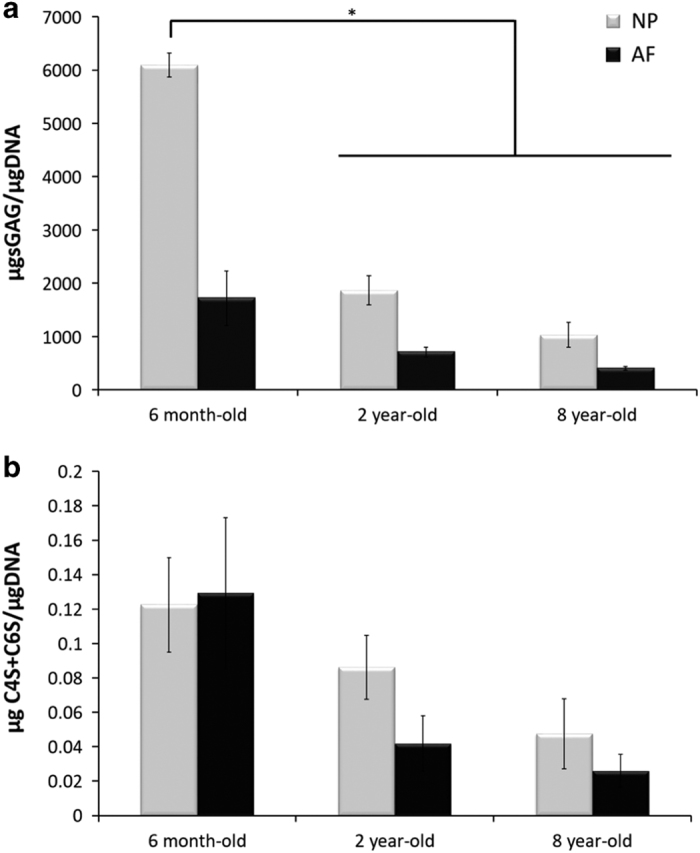
Age and tissue-related changes of (**a**) total sGAG content of bovine IVD quantified by the DMMB assay and (**b**) sulfated CS content quantified by HPLC. Data were normalized to the DNA content and are represented as the mean±s.e.m. (*n*=5). * represents significant differences at *P*<0.05.

**Figure 6:**
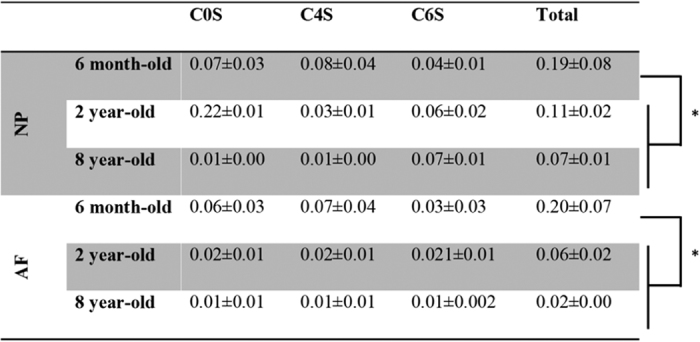
Quantities of C0S, C4S and C6S disaccharides (μg/μg of DNA) in 6-month, 2-year and 8-year-old bovine IVD tissues. Data were normalized to the DNA content and are represented as the mean±s.e.m. (*n*=5); * represents significant differences at *P*<0.05 for C0S, C4S, C6S and the total CSs.

Similarly, an iterative decrease of the sulfated glycosaminoglycan (sGAG) content was revealed after quantification by the dimethylmethylene blue (DMMB) assay in both NP and AF tissues upon ageing (Figure 5a). The sGAG content was higher in NP tissue than in AF tissue for each age-group (*P*<0.05). The ECM of 6-month-old NP tissue contained six times more sGAG than 2-year-old NP ECM and three times more sGAG than 8-year-old NP ECM, respectively. A comparable but less pronounced trend was observed for the AF ECM. The sulfated CS disaccharide content, obtained by HPLC analysis, showed a similar profile to that observed for the total sGAG content with an age-related decrease in the CS content. However, this trend was not statistically significant (Figure 5b). The quantities of specific disaccharides chondroitin-0-sulfate (C0S), C4S and C6S varied with ageing. Quantification of the data is presented in Figure 6 as μg of disaccharide per μg of DNA for normalization. The total CS disaccharide content decreased in NP tissue from 0.19±0.08 μg of CS/μg of DNA in 6-month-old tissue to 0.066±0.01 μg of CS/μg of DNA in 8-year-old tissue (Figure 6).

**Figure 7:**
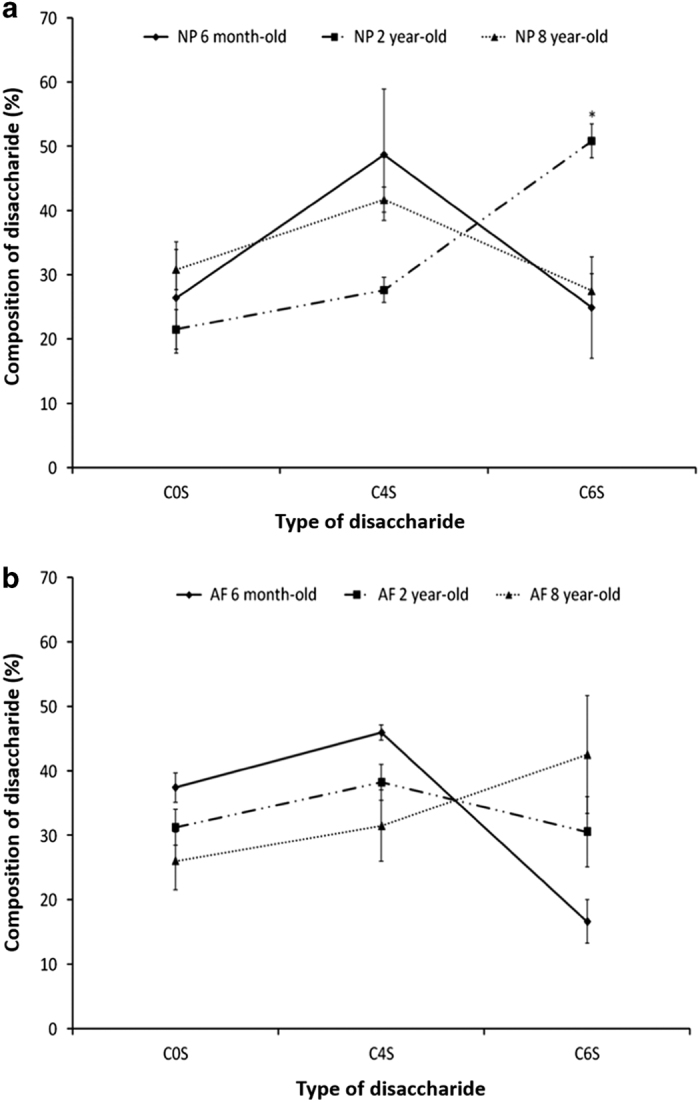
Percentages of disaccharides C0S, C4S and C6S in 6-month-old, 2-year-old and 8-year-old bovine (**a**) NP and (**b**) AF tissues. Data were normalized to the DNA content and are represented as the mean±s.e.m. (*n*=5); * represents significant differences at *P*<0.05.

In AF tissue, the total CS disaccharide content dropped from 0.20±0.07* *μg of CS/μg of DNA in 6-month-old tissue to 0.02±0.01 μg of CS/μg of DNA in 8-year-old tissue. NP and AF tissues do not display the same changes in CS composition upon ageing (Figures 7a and b). Although the percentage of C0S in the tissues remained constant with age for both tissue-types, significant differences were noted in C4S and C6S disaccharide profiles. At 6 months and 8 years, NP tissues showed a comparable profile with lower percentages of C0S and C6S and higher proportions of C4S disaccharides (Figure 7a). NP ECM in 2-year-old tissue exhibited an inverse profile with a higher percentage of C6S disaccharides and a lower percentage of C4S disaccharides. A progressive switch of the sulfation profile was noted in the AF from 6 months to 8 years. Eight-year-old AF ECM contained more C6S disaccharides than C4S disaccharides. However, for this tissue, the variations in the percentages of disaccharides between the different age-groups were not significantly different (Figure 7b).

## Discussion

During the degeneration and ageing of the IVDs, PG depletion occurs, which can be induced by IL-1β or other catabolic factors.^43,45,46^ Aggrecan is the main PG of IVD tissue, substituted with many GAGs, among which CSs are the most abundant.^43^ During the degeneration and ageing processes, the size and number of CSs carried by aggrecan decrease, which influences the IVD at both structural and biological levels.^15,32,47^ In this study, the behavior of GAGs, specifically CSs, was investigated in an ageing bovine model. This model was chosen because of its similarity to human IVDs due to its similar size,^48^ loss of notochordal cells,^49^ gene expression^50^ and GAG behavior.^49^

Miyazaki *et al.*^49^ attributed the similarity between human and bovine GAG behavior to the absence of notochordal cells within the tissue. These cells were shown to greatly influence the production of GAGs. Notochordal cells were not observed in six month-old tissue (Figure 3(1) (a)). As a consequence of the disappearance of notochordal cells, the quantity of sGAG produced decreases and an increase of sGAG degradation enzymes occurs, resulting in an imbalance in anabolic and catabolic activities.^49^ This phenomenon results in the formation of a less hydrated and flexible tissue as a function of age.^4^ These characteristics were observed in the ageing bovine model evaluated here (Figure 3). At a histological level, a decrease of GAG staining was noted between 6-month-old and 2-year-old tissues, which suggests a decrease of anabolic and/or an increase of catabolic activities by IVD cells with ageing.

Close observation of the total sGAGs including CS, HS, KS and DS revealed an iterative decrease in their quantity upon ageing (Figure 5a). CSs, the most abundant sGAG within the IVD tissue, were shown to play essential structural and biological roles in the IVD.^43^ Their polyanionic nature allows the retention of water within the tissue ^51^ as well as interaction with growth factors (GFs) and cytokines.^47^ These properties confer specific roles to CSs in tissue development, remodeling and pathology.^52–54^ The CS composition was specifically assessed by HPLC. An iterative decrease in total CS content was observed upon ageing (Figure 6). However, this decrease was less marked than the decrease observed in the total sGAG content. This observation suggests that not only CSs but also the overall sGAG content, including CS, HS, KS and DS, was affected by the phenomenon of ageing. An inversion of the sulfation profile occurs with maturity (from 6 months to 2 years) with a higher level of C4S in 6-month-old tissue and a higher level of C6S in 2-year-old tissue (Figure 7a). The ECM of the NP in 8-year-old tissue displayed a similar trend to that of 6-month-old tissue. These variations in the CS disaccharide composition can be attributed to changes in the mechanical stresses on the discs (for example, bending, flexion), which has been shown to modify the CS sulfation pattern.^55^ This variation in the sulfation pattern is obtained by the successive actions of sulfotransferases and sulfatases ^56^ that allow rapid changes in the sulfation pattern and consequently in cell–cell and cell–ECM communication.^32,56,57^ GFs and cytokines interact with specific sulfated disaccharides in a time- and gradient-dependent manner.^14,58^ The modification of CS disaccharides also suggests that there is a role for these molecules in the regulation of GF and cytokines during the development and ageing processes of the IVD. The AF ECM of 8-year-old tissue contained less C4S disaccharides and more C6S disaccharides relative to both the 6-month and 2-year-old tissue (Figure 7a). The results of this study highlight the variations in the composition and content of CSs, which occur during IVD ageing. A significant decrease in the overall CS content is observed in this study (Figure 6). The biosynthesis of CSs is performed by the polymerization of GlcA and GalNAc catalyzed by different enzymes.^39,59^ XT-I and GT-I are considered to play key roles in the synthesis of CSs.^36,38,42^ The behaviors of both enzymes were evaluated by immunohistochemistry upon ageing. XT-I and GT-I were detected in both AF and NP tissues (Figure 3 and Figure 4). Decreases of XT-I and GT-I occurred upon ageing, and they were undetected in 8-year-old tissue. Therefore, a correlation between the behavior of CSs and their synthetic enzymes can be made. The control of CS synthesis remains unclear. The regulation of their synthesis occurs at gene, enzymatic and turnover levels.^57,60^ Growth factors such as BMP-2 and transcription factors such as TonEBP (tonicity sensitive transcription factor) and hypoxia inducible factor one (HIF-1) and HIF-2 (hypoxia sensitive transcription factors) are implicated in the rate-limiting nature of the enzymes involved in the synthesis of CSs.^35,43,61,62^ In addition, successive phosphorylation and sulfation phenomena take part in the enhancement or repression of the synthesis of CSs.^63,64^ These phenomena are likely disrupted during the ageing and degeneration of the IVD. Further investigations on their impact on CS synthesis and CS enzymes including the expression of sulfotransferases and sulfatases are required to elucidate a more comprehensive understanding of IVD pathologies. It is important to note that bovine IVDs have been shown to present different mechanical properties than human discs.^65,66^ Therefore, it is essential to specifically characterize the profile of CSs in humans to validate the findings of this study.

## Conclusions

Significant changes in GAG composition during disc ageing were highlighted in this study. The content and composition of CSs, the main GAGs present in the IVD, and of key enzymes for the synthesis of CSs, were affected during the ageing process. These changes provide an appreciation of the potential impact of CS composition on disc biology. Better understanding of disc biology plays an essential role in the development of different therapeutic approaches for disc regeneration and repair.^67^

## Materials and methods

### Materials and reagents

Anti-XT-I antibody was purchased from Santa Cruz Biotechnology Inc. (Santa Cruz, CA, USA). Anti-GT-I antibody (anti-B3GAT1) was purchased from Abnova (Heidelberg, Germany). Prolong Gold antifade, secondary antibodies AlexaFluor 555 mouse anti-goat and AlexaFluor 555 donkey anti-mouse, 4’,6’-diamidino-2-phenylindole dihydrochloride (DAPI) and Quant-iT PicoGreen dsDNA reagent were purchased from Life Technologies (Dublin, Ireland). All other materials and reagents were purchased from Sigma-Aldrich (Dublin, Ireland) unless otherwise stated.

### Tissue collection and preparation

Bovine caudal IVDs from 6-month-old, 2-year-old and 8-year-old animals were collected directly after killing from the local slaughter house (*n*=5 for each age-group). Soft tissues surrounding the IVDs (muscles and ligaments) were removed. Five discs without the cartilage end-plate for each age-group were then fixed in 4% neutral buffered paraformaldehyde (PFA) overnight at 4 °C for histology, while NP and AF tissues from five different animals were digested for GAG biochemical analysis. Each IVD was sectioned transversally through the center, and NP tissue and AF tissue were harvested from both halves.

### Histology

After fixation, discs were washed three times with 1× PBS and infiltrated with 20% sucrose overnight at 4 °C. Specimens were flash-frozen in liquid nitrogen-cooled isopentane and 5 μm frozen sections were cut transversally on a Leica CM 1850 cryostat (Laboratory Instruments & Supplies Ltd., Ashbourne, Ireland) and stored at −20 °C until use. Each IVD was sectioned transversally through the center.

### Safranin O/fast green staining

Sections were washed three times with 1×PBS before staining. The sections were then stained with Safranin O (proteoglycans staining) and FCF (collagen staining). After dehydration through a series of ethanol and xylene baths, sections were mounted with distyrene plasticizer xylene (DPX) mounting solution. After drying, slides were viewed under a light microscope (Olympus Fluorescence Microscope, Middlesex, UK).

### Immunohistochemistry

Immunohistochemistry was performed for three different antigens: XT-I detected by goat anti-XT-I (anti-XYLT1) antibody (Santa Cruz Biotechnology Inc., Santa Cruz, CA, USA), GT-I detected by mouse anti-GT-I (anti-B3GAT-1) antibody (Abnova, Heidelberg, Germany) and C6S and C4S detected by the CS-56 antibody. For the detection of XT-I and GT-I, antigen retrieval was required by digestion with proteinase K at 20 μg ml^−1^ (30 U mg^−1^) for 5 min at 37 °C. After digestion, slides were washed three times in 1× phosphate-buffered saline (PBS) buffer solution. For all staining, sections were blocked with 5% goat serum in 1×PBS for 1 h before overnight incubation at 4 °C with the primary antibodies diluted at 1/200 in 0.1% serum. Negative control sections were incubated without primary antibody. After five washes with 1×PBS with 0.05% Tween 20 for 5 min each, the secondary antibody (AlexaFluor 555 mouse anti-goat for anti-XT-I antibody and AlexaFluor 555 donkey anti-mouse for anti-GT-I and CS-56 antibodies; Life Technologies)) diluted at 1/1000 in 1×PBS was incubated for 1 h at room temperature (RT) with the sections. The slides were then washed five times with 1×PBS with 0.05% Tween 20 for 5 min each before being counterstained with 4’,6’-diamidino-2-phenylindole dihydrochloride (DAPI) (Life Technologies) diluted at 1/2500 in 1×PBS for 10 min at RT. The sections were washed three times with 1×PBS with 0.05% Tween 20 before mounting with Prolong Gold antifade (Life Technologies). The slides were cured at 4 °C in the dark for 1 day before imaging. Imaging was performed using an inverted epifluorescent microscope (Olympus IX81, Mason Technologies, Dublin, Ireland). Five images per slide were taken and analyzed.

### Image analysis and fluorescence intensity quantification

Quantification of the fluorescence intensity of the digital images was performed using ImageJ software (National Institutes of Health, USA). The intensity of the fluorescence was measured from five different images for nucleus pulposus and annulus pulposus tissues (*n*=3). The intensity of the fluorescence values was then normalized to the cell number. For each cell-group, five images were quantified (*n*=5). The average fluorescence intensity quantification from the three sets of cells with the s.e.m. was represented.

### GAGs quantification

After isolation, NP and AF tissues were digested overnight at 56 °C in proteinase K at 0.5 mg ml^−1^ before further analysis.

### Biochemical quantification of the total sGAGs by DMMB

The sGAG content was quantified by the dimethylmethylene blue (DMMB) assay. Chondroitin 4-sulfate from bovine trachea was used as a standard, and the absorbance was measured at 535 nm. DNA quantification for the sGAG content normalization was performed using the Quanti-iT PicoGreen assay (Invitrogen, Dublin, Ireland) according to the supplier’s instruction.

### Biochemical quantification of CSs by HPLC

One hundred microliters of the proteinase K-digested NP, AF or cartilage tissue was filtered through a 3 kDa molecular weight cut-off (MWCO) centrifugal filter with one hundred microliters of HPLC-grade water according to the manufacturer’s instructions. All centrifugal filters were washed with HPLC-grade water prior to use, and water used throughout the procedure was of HPLC-grade. The retentate was eluted from the filter with 50 μl of water and 390 μl of digestion buffer (50 mm Tris-HCl, 60 mm sodium acetate, pH=8.0), and 10 μl of chondroitinase ABC (ChABC; 100 mU) was added. The mixture was then digested at 37 °C for 3 h with gentle agitation (300 r.p.m.). The digested mixture was then immediately filtered as above (3 kDa MWCO), and the lower molecular weight filtrate was dried in a vacuum centrifuge (~2 h). The dried and digested samples were then stored at −20 °C until analysis. For analysis, digested samples were dissolved in 200 μl of water, and 10 μl of the sample was injected on an Alliance 2695 instrument (Waters, Dublin, Ireland). Briefly, chromatographic separations were carried out on an Ultratech 5ODS C18 (250 mm×4.6 mm) (HPLC Technology, Inc., Cheshire, UK) or a Synergi column (250 mm×4.6 mm, 4 μm, 80 Å) (Phenomenex Inc., Torrance, CA, USA) at 25 °C at a flow rate of 1.1 ml min^−1^, and the eluate was monitored at 232 nm absorbance on a Waters 2489 UV–Vis detector. The mobile phase consisted of (A) 1 mm or 2 mM aqueous tetrabutylammonium bisulfate (TBAB) and (B) 1 mm or 2 mm TBAB in a 2:1 mixture of acetonitrile and water, with starting conditions of 80% A and 20% B. A gradient of 20–65% B was applied over 7 min, followed by holding at 65% B for 5 min and finally returning to 20% B after 12.5 min. The system was re-equilibrated at 20% B for 10 min before the next sample injection. The disaccharide content of each sample was identified by comparison to appropriate disaccharide reference standards (ΔC4S, ΔC6S and ΔC0S) under the same HPLC conditions as the sample and quantified by comparison to the appropriate standard curve generated by the injection of known concentrations of the standards.

### Statistical analysis

Statistical analysis was performed using GraphPad Prism, Version 5 (USA). Data were compared using one-way analysis of variance (ANOVA) followed by a Tukey comparison test. Values were considered to be significantly different with a *P*<0.05.
